# Parenting Stress in Mothers of Children with Permanent Hearing Impairment

**DOI:** 10.3390/children10030517

**Published:** 2023-03-06

**Authors:** Grazia Isabella Continisio, Domenico D’Errico, Silvia Toscano, Nelson Mauro Maldonato, Raffaella De Falco, Francesco Nunziata, Angelica Rodio, Antonio Casarella, Valeria Del Vecchio, Anna Rita Fetoni, Rita Malesci

**Affiliations:** 1 Department of Neuroscience, Reproductive Science and Dentistry, Università degli Studi di Napoli Federico II, Via Pansini, 5, 80131 Naples, Italy; 2 Section of Audiology, Department of Neuroscience, Reproductive Science and Dentistry, Università degli Studi di Napoli Federico II, Via Pansini, 5, 80131 Naples, Italy; 3 Section of Pediatrics, Department of Translational Medicine Science, Università degli Studi di Napoli Federico II, Via Pansini, 5, 80131 Naples, Italy

**Keywords:** parental distress, permanent hearing impairment, hearing loss, early detection, intervention program, early rehabilitation

## Abstract

Permanent childhood hearing impairment (PCHI) represents the most frequent sensory pathology at birth. PCHI has a relevant psychological impact on the life of both the affected children and their families. Thus, the aim of this work is to explore the degree of parental distress felt by mothers of a deaf or hard-of-hearing child, to determine if this stress is associated with variables related to the children’s health (e.g., the severity of hearing loss, presence of other conditions, difficulty with treatment options, difficulty with rehabilitation) or family characteristics such as socio-economic and educational status. The study used the Parenting Stress Index–Short Form (PSI-SF) questionnaire administered to mothers. The results were analyzed in relation to variables such as parents’ education level, number of children, severity of hearing loss, presence of other chronic conditions, presence of cognitive delay, familiarity with hearing loss, time of diagnosis, use of prosthetics, and start in a rehabilitation program. The data indicate a correlation between maternal stress levels and low-educational levels, as well as the presence of congenital infections and cognitive delay. These results highlight the need for a comprehensive physical and psychological approach for hearing-impaired children, as stress factors can affect the adherence to effective rehabilitation.

## 1. Introduction

Permanent childhood hearing impairment (PCHI) is the most common form of sensory pathology at birth, with an incidence rate of approximately 0.5–1.5/1000 in newborns [1]. PCHI has a significant psychological impact on both the affected children and their families, and can result in challenges with language and communication, decreased academic and vocational achievements, and mental health problems [2].

Although early diagnosis and intervention have mitigated some of the handicaps associated with hearing loss, the families of affected children still face significant challenges, with up to 40% of these children also having associated cognitive and neuro-motor difficulties [3,4].

At present, deafness cannot be diagnosed before birth, and can only be suspected in the infants of deaf parents, as the genetic factor is relevant, or in women who have contracted viral infections during pregnancy such as cytomegalovirus, rubella, toxoplasmosis. Otherwise, in infants without risk factors, the parent may not suspect that the child may have a hearing disorder. The Joint Committee on Infant Hearing (JCIH) therefore recommends that all neonates undergo hearing screening tests within the first month of life and receive a diagnosis by 3 months of age, with treatment and interventions starting by 6 months of age [2]. Early diagnosis allows for timely fitting of hearing aids or cochlear implants, helping to avoid the negative consequences of hearing deprivation, and allowing the children with PCHI to grow up with normal developmental index scores, both in terms of academic and socioeconomic progress as well as in their emotional and psychological integrity.

Newborn hearing screening and an early diagnosis of PCHI create an opportunity, but they do not guarantee optimal outcomes because they are only the first steps of a long habilitation program to promote normal language development. Indeed, the literature highlights the benefits of early family involvement in the habilitation program to obtain better results.

The family is the natural environment in which a child grows. As a result, professionals encourage parents to take an active role in the intervention process and develop programs for the whole family to help the child achieve the maximum possible benefit.

There is no doubt that the family of a child suffering from a chronic disabling disease or a disability often faces complex situations or has to take difficult decisions, reporting high levels of parenting stress [5].

Various studies have shown the influence of stress on parents who have deaf or hard-of-hearing children in terms of both practical and emotional involvement [6,7,8,9,10,11,12]. On the other hand, variables such as poorer social-emotional functioning and the language ability of the child are related to higher stress levels in parents more than the degree of hearing loss [13].

Thus, information deriving from an internationally validated quantitative stress test of parents, such as the Parenting Stress Index–Short Form (PSI-SF), could be a reliable way to help these families. The aim of this work is to explore the degree of parental distress among deaf or hard-of-hearing children’s mothers and to understand whether it is associated with variables related to the children’s health (severity of hearing loss, presence of other associated diseases, difficulty in treating deafness with prostheses or cochlear implantation, difficulty in managing the rehabilitation process) or with the characteristics of the family such as socio-economic and educational level, and support from the extended family. 

## 2. Materials and Methods

### 2.1. Sampling and Eligibility

A retrospective cross-sectional observational study was carried out at the Unit of Audiology of the University Hospital Federico II, the Regional Reference Center for Early Diagnosis of Deafness of the Campania Region, between June 2019 and June 2021. 

The study recruited participants from the families of children in therapeutic follow-up, and the sample consisted of 82 adult mothers and 82 children (56 males and 26 females). During the study period, 23 adult mothers declined to participate. The parents of all children involved in the study were at least 18 years old and gave their informed consent to the use of anonymized data.

All mothers included in this study received a diagnosis of hearing loss for their child at our outpatient clinic. 

The severity of hearing loss was classified according to ASHA 2015 guidelines: normal hearing indicated as 15 decibels in hearing level (dB HL) or better, slight HL as 16 to 25 dB HL, mild HL as 26 to 40 dB HL, moderate HL as 41 to 55 dB HL, moderately severe HL as 56 to 70 dB HL, severe HL as 71 to 90 dB HL, profound HL as 91 dB HL or worse.

As shown in Table 1, the evaluation of all families included an accurate investigation of both the children (age, clinical history, comorbidities, age of diagnosis and rehabilitation) and the families (age of mothers, level of education, employment, presence of siblings).

We classified the age of the children at time of diagnosis (<6 months, 7–12 months, 13–24 months, >25 months) and at time of the parental stress evaluation (<24 months, 25–36 months, >36 months). We also included the strategy of hearing rehabilitation with cochlear implant. 

Possible conditions related to hearing loss were also collected, such as hereditary hearing impairment and confirmed genetic diagnosis of mutation of connexin 26, congenital infections by TORCH complex, low birth weight less than 1800 g, the presence of craniofacial malformation, confirmed syndromes, and cognitive delay. Cognitive assessment was performed using either the Wechsler Preschool and Primary Scale of Intelligence–Third Edition (WPPSI-III), and the criterion for determining the cognitive delay was an IQ score < 70–75.

Regarding the investigation of the families, the age of the mothers was classified into three ranges (<25, 26–34, ≥35 years of age). The level of education was obtained by the highest finalized degree and categorized into two groups, considering the cut-off of 8 years of school education: primary/middle school (≤8 years) and high school/degree (>8 years). We also determined whether the mothers were employed or homemakers.

### 2.2. Ethical Approval 

This research project was approved by the University of Naples Federico II Ethics Committee (protocol number 56/18 on 26 March 2018).

### 2.3. Instrument

A validated international level questionnaire was administered to measure the degree of stress. The questionnaire, called PSI-SF, was designed by Richard R. Abidin [14] and is based on the Parenting Stress Index (PSI) which is currently used to assess the relative stress in the parent–child relationship. Child characteristics in the full-scale include distractibility/hyperactivity, adaptability, reinforcement of the parent, demandingness, mood, and acceptability. The PSI is self-administered and includes a score in three areas: parental distress, dysfunctional parent–child interaction, difficult child, as well as a summary score defined as total stress [15].

This tool is based on the assumption that high levels of stress within the parental system are critical for the emotional/behavioral development of the child, as well as the development of a positive relationship with the parents. The PSI-SF has been standardized with parents of children aged 1 to 12 years and includes 120 items, with the last 19 Life Stress Items (LS–stressful life events), ranging from 102 to 120, being optional.

The PSI is used for the early detection of characteristics that can impair the normal development of the child. The aim of the PSI is to identify children with emotional or behavioral disorders and parents with a risk of dysfunctional parenting. The tool is based on the assumption that parental stress depends on the specific characteristics of both the child and the parent but also on a series of situations closely related to the role of the parent.

The Italian validation of the test only includes its short form (PSI-SF) [14], as it is a convenient and immediate instrument.

The PSI-SF questionnaire assesses three key sources of stressors in the parent–child relationship, including the characteristics of the child, the parent, and environmental events. The Short Form of the questionnaire consists of 36 items, divided into 3 subscales:

1. Parental distress (12 items), reflecting personal factors that contribute to stress in the role of the parent;
2. Parent–child dysfunctional interaction (12 items), examining the parent’s perception that the child is not responding to their expectations as well as the lack of reinforcement in the parent–child relationship;
3. Difficult child (12 items), considering key characteristics of the child that may make them difficult to manage, including traits related to their temperament and acquired patterns of defiance, disobedience and demanding behavior.

In addition to the investigated items, a correlation between the mother’s stress level and depression or sadness, meaning the extent of involvement of the mother’s life could be a hint for further supporting measures.

Additionally, a defensive response score can be calculated to assess the tendency to present a more favorable self-image, minimizing problems or stress in the parent–child relationship.

The scores for the three subscales and the total distress score are calculated from the completed questionnaires.

### 2.4. Statistical Analysis

The data were analyzed, taking into account potential confounding variables such parents’ level of education, number of siblings, severity of hearing loss, presence of other chronic diseases, presence of cognitive and neuropsychomotor delays, familiarity with hearing loss, time of diagnosis, prosthesis and start on a habilitative program. Differences between groups were evaluated by the Fisher’s exact test, when appropriate, for categorical variables and by the non-parametric Mann–Whitney test for continuous variables. The χ^2^ test and was performed to determine significant differences in proportions or percentages between the two groups. χ^2^ test with Yates continuity correction was used where the χ^2^ test was not appropriate. In addition, the binomial test was performed to compare two mutually exclusive proportions. Any significant difference between the two means was evaluated by the Student’s *t*-test. Multiple comparison chi-square tests were carried out to highlight any significant differences among the percentages. The statistical significance level was set at *p* < 0.05. Data processing was performed using SPSS software Edition. 11.

## 3. Results

The demographic data of the enrolled patients, both relative to parents and children are reported in Table 1.

Table 1 also describes the clinical characteristics of hearing impaired children based on severity of hearing loss, age of diagnosis, types of hearing devices (hearing aids or cochlear implant), and presence of associated comorbidities. In the sample, the male sex is particularly represented (*p* = 0.029 C), highly educated mothers (*p* < 0.001 Z), profound hearing loss (*p* < 0.001 C) cognitive delay, low birth weight, syndromes and a rather delayed time of diagnosis (*p* < 0.001 C).

Statistical tests were performed for each variable. The most common mother’s age was between 18 and 37 years. They had a “High School” education level and they were more often housewives. The proportion of housewives was higher among mothers with low-educational level (94% versus 51%; *p* < 0.05). The age of the children was more than 36 months (42%) when mothers completed the Parenting Stress Index questionnaire. With regard to the clinical features of children with hearing loss, the time of diagnosis was obtained early and before 6 months of age (39%) and the principal associated clinical conditions were cognitive delay (43%), congenital infections (12%) and syndromes/dysmorphisms (39%). In terms of disease severity, the most frequent degree was “profound” (53.6%). The rehabilitation modality for children with hearing loss consisted of 52 with hearing aids (63.41%), and 30 with cochlear implants (37%).

Table 1 and Table 2 provide information regarding the level of stress in the three subscales and of overall stress. The areas of stressors related to the characteristics of the child were more often difficult child (38%) and dysfunctional parent–child (29%). The subscale of parental distress (19.5%) had a very slight difference as compared to the total population but about twice the increased stress in the other dimensions.

In particular in Table 1, the overall results for each domain are represented, while Table 2 shows the information with a univariate analysis considering the potentially 12 confounding variables. Remarkably, the subscales mostly affected were the dysfunctional parent–child interaction (29%) and the difficult child (38%). 

The subscale of parental distress (19.5%) had a very slight difference as compared to total population but about twice the increased stress in the other dimensions.

As reported in Table 2, the presence of cognitive/neuromotor impairment, the mother’s low level of school education, and the presence of infections at birth represent risk factors for parental stress. The cognitive/neuromotor impairment is a major comorbidity affecting maternal stress. The influence of cochlear implant on parental stress was also evaluated.

Comparing the PSI of mothers of children with different hearing rehabilitation strategies, the mothers of children not rehabilitated with cochlear implant achieved worse results and their risk of stress was three-fold increased over mothers of children treated with cochlear implant (odds ratio 3.29, *p* values 0.05). Moreover, the school education level also influenced the impact of stress. A decreased education level of less than 8 years had a negative statistical significance on parental stress. (odds ratio 0.17, *p* value 0.01).

## 4. Discussion

In our study, we aimed to assess the stress condition and coping skills in mothers of deaf or hard-of-hearing children following the confirmation of hearing loss, fitting with hearing aids and/or cochlear implantation, and access to an intervention program. 

We found that 32% of our sample of mothers reported particularly high stress levels. Families with children who had comorbidities associated with hearing loss were at higher risk of stress due to their multi-problematic socio-family structure. Interestingly, we observed that risk factors for general stress included the presence of cognitive delays, low levels of parental education and of congenital infections. Conversely, a high level of education in the mother and a cooperative family environment were protective factors against stress [16].

The presence of a child with special needs intensifies the challenges of parenting. Parents of children with emotional and/or developmental disabilities must not only face the typical stressors of parenting but also a host of stressors unique to their child’s care. A pervasive stress affecting families is the failure of society to understand and accept their children, which often leads to focus on the negative aspects of the disability. This complex social and familiar situation can cause frustration, particularly for the mother, as our results suggest, and the world around them may misinterpret that anger as “bad parenting”. These conflicts may make parents feel estranged from a world where normality and health are viewed as the optimum. 

For these reasons, parents of children with special needs often report feelings of anxiety, depression, loneliness and hopelessness. Several studies have highlighted stressors for parents, including difficulties in accepting and adjusting to their child’s disability, limited or absent accessible information about their child’s disability, financial demands for necessary medical equipment and care, time-management conflicts, and poor access to the appropriate services to relieve their caretaking activities. The added stress confronting this population is well studied [17,18,19,20].

Hearing loss can affect a child’s language acquisition and development, which naturally occurs through significant interactions between parents and children [12]. Early diagnosis and intervention are critical steps towards the proactive management of these children due to the critical window available for auditory brain development and language acquisition [14]. Recent evidence indicates that providing comprehensive intervention services by 6 months of age can enable many children with sensorineural hearing loss to achieve language abilities similar to their hearing peers [21]. The presence of deafness in a family affects all aspects of family life. Understanding the impact on family life is fundamental to involve all components of the family system in early intervention. The current model of early intervention with deaf or hard-of-hearing children emphasizes parental self-efficacy and involvement [22].

The current guidelines of the Joint Committee on Infant Hearing (JCIH) emphasize the essential role of parental training in the auditory, speech and language rehabilitation of hearing-impaired children. Parents who participate in intervention programs have been found to communicate better with their children and contribute more to their child’s progress compared to parents who do not participate. In this family-centered scenario, the presence of a multidisciplinary team has a great impact on child outcomes [9]. Parents of newborns with hearing loss identified by Universal Newborn Hearing Screening (UNHS) programs require educational support shortly after confirmation and contact with other affected families [23,24,25,26].

Several studies have evaluated the impact of parental involvement in intervention program and the impact of cofounders and comorbidities. Calderon et al. [27] retrospectively analyzed characteristics of 28 families who participated in the same early intervention program. They found that late identification results in families spending limited time in early intervention program. Therefore, professionals should guide parents to take an active role in the intervention process and develop programs for the whole family in order to lead the child to achieve the maximum possible benefit.

In addition to distal variables such as socioeconomic status [28], it has been demonstrated that variables related to the proximal environment, such as parental sensitivity, quantity and quality of parental language, home literacy environment, significantly contribute to the prediction of child language development [29].

What makes it possible to assess the degree of family involvement is the Parenting Stress Index–Short Form (PSI-SF), an internationally validated tool that explores various areas of discomfort and coping, related to parent–child interaction, parental distress and behavior [30].

High levels of parental stress can have detrimental effects on a child’s development, reducing the parenting effectiveness in habilitative program based on family involvement. Increased parental stress has been found to influence parent–child interactions, contributing to more controlling and less responsive parenting behaviors and inappropriate linguistic input. Therefore, the assessment of parenting stress is fundamental to avoid undermining the effectiveness of these early interventions [31,32].

To date, several studies have examined the rates of parenting stress among parents of young deaf children, but contradictory findings have emerged due to differences in measurement. According to these studies [7,8,9,10,11] factors that contribute to parenting stress in parents of deaf children include age at diagnosis, degrees of hearing loss, language abilities, mode of communication (spoken language or sign language), child behavior problems, perceived social support, cochlear implants, additional disability with poorer language skills, or higher rates of behavior problems [6]. Conversely, other studies have not found higher levels of stress among parents of deaf or hard-of-hearing children [33,34,35,36,37,38]. According to Dammeyer et al. [34], it is not the hearing loss itself but child-related characteristics, such as additional disabilities and behavioral, emotional, and social difficulties, that negatively impact parenting stress. Fitzpatrick et al. [33] found that parents of children identified early with unilateral and mild bilateral hearing loss and normal performance in multiple domains including language and behavior, did not report higher stress levels than parents of children with typical hearing [31,32,33,34,35,36]. 

In our research we focused on mothers as the primary caregivers and assessed their parenting stress levels. Previous studies have reported comparable levels of parenting stress among mothers and fathers of children with disabilities [39,40], as well as mothers and fathers of deaf or hard-of-hearing children [10,34,35,36,37,38,39,40,41,42,43]. Consistent with several studies, our sample indicates that the mother’s level of education affects the child’s outcomes: mothers with a higher level of education report greater levels of active participation in the rehabilitation process. We postulate that the mother’s cultural level allows for higher expectations of their child’s performances. Other factors more closely related to the child also impact the degree of parenting stress, such as the child’s age, type of device used, and presence of additional disabilities. The choice of cochlear implantation seems to be correlated with a decrease in parenting stress and in a reduced risk associated with low maternal education, as reported in Table 3. Mothers of children with cochlear implants appear to be more involved than mothers of children rehabilitated with hearing aids, possibly due to the different rehabilitation path followed and the mother’s sense of responsibility for the therapeutic choice.

Surprisingly, the decision to undergo cochlear implant surgery appears to decrease parenting stress, potentially due to the improvements achieved or the belief of the parents that they have done everything possible for their child. Horsch et al. [37], found that parents of children with hearing loss who underwent cochlear implantation reported stress levels comparable to those of parents of children without hearing loss. In contrast, Spahn et al. [38] reported higher parental stress among parents of children with cochlear implant compared to those with hearing aids, particularly during the period surrounding implantation. Despite the pivotal role of the healthcare team’s preparation in the level of acceptance of the child’s rehabilitation, the family’s decision-making process is complex and involves constant and intense moments of reflection. The parents, particularly mothers, experience anxiety before the choice of cochlear implantation. During the preparation phase for decision-making, it is crucial to work with the parents on their expectations and feelings of accountability, guilt, and fear of possible surgical risks to reduce anxiety and frustration after the decision has been made. Children who receive cochlear implant generally have greater hearing losses than those who use hearing aids, confirming there is no association between the child’s degree of hearing loss and parental stress levels. Another interesting aspect is that, in the absence of cognitive delay, the stress levels of parents of children with low-hearing loss is practically the same as the stress levels of parents with normal-hearing children. Therefore, it must be taken into consideration that the findings of this study are similar to previous research studies that have concluded that associated developmental pathologies, such as cognitive or behavioral disorders may contribute to parenting stress beyond just the audiological domain [8,35].

In terms of age, parents of younger children appear to be more involved in the enabling process and benefit more emotionally from actively participating in the process. Additionally, communication expectations are minimal during infancy, leading to lower stress levels that may increase as children grow and communication difficulties and behavioral problems arise [8,44].

In conclusion, our data shows a correlation between maternal stress levels and the mother’s level of education and comorbidities, such as cognitive and motor difficulties included in the child difficulty domain of the Parenting Stress Index–Short Form questionnaire. These results highlight the need for an integrated physical and psychological approach to all hearing-impaired children, as stress factors can affect the adherence to proper rehabilitation.

### Limitations

The study we conducted has certain limitations. The sample size used in our study was relatively small in comparison to other studies that have examined the psychometric properties of PSI-SF. This has limited the achievement of the study objective. The statistical analysis was also limited by the small sample size, which did not allow for the execution of more effective analyses, and confounding factors such as child gender, mother’s age, and child age were not excluded.

In addition, this study highlights the need for further research, including fathers’ education, their employment status, marital status, and income, and their potential effects on PSI scale and other psychological outcome parameters.

Therefore, the current data should be interpreted as preliminary findings to encourage future research on the psychometric properties of PSI-SF with larger samples and similar demographic compositions within a multicentric study. However, despite these limitations, the study has demonstrated that the clinical management of hearing-impaired children should include psychological support for parents. Parenting stress plays a fundamental role in compromising maternal involvement in the early intervention of hearing-impaired children especially when they are rehabilitated with hearing aids as opposed to a cochlear implant.

## Figures and Tables

**Table 1:** Characteristics of subjects enrolled.

Characteristics	Number	%
**Child Gender**		
Male	56	68
Female	26	32
**Child age**		
<24 months	19	23
25–36 months	28	34
>36 months	35	43
**Mother’s school education**		
Primary/middle school	35	43
High school/degree	47	57
**Mother’s job**		
Housewife	57/82	69
Employed	25/82	31
**Mother’s age**		
18–25 years	8	10
26–34 years	39	47
35–37 years	35	43
**Other brothers and sisters**		
Yes	42	51
No	40	49
**Hearing loss severity**		
Mild	22	27
Severe	16	19.5
Profound	44	53.6
**Presence of cognitive delay**	35	43
**Hearing loss familiarity**	17	22
**Low birth weight**	36	45
**Positivity for connexin 26 mutation**	19	23
**Congenital infections**	9	12
**Syndromes/craniofacial malformation**	32	39
**Time of diagnosis**		
<6 months	32	39
7–12 months	26	32
13–24 months	12	15
>25 months	8	10
**Cochlear implant**	30	37
**PSI subscales (Values > 85 centile)**		
Parental distress	16	19.5
Dysfunctional parent–child interaction	24	29
Difficult child	31	38
Overall stress	26	32
Defensive response	16	19

B = exact binomial test, C = multiple comparison χ^2^ test, Z = Z-test.

**Table 2:** Univariate associations between child’s and family’s characteristics with overall stress.

Variables	Stress > 85 Centile Number (%)	Odds Ratio (IC 95%)	*p* Values
**Child Gender**			
Male (*n* = 56)	17 (30)	0.8 (0.3–2.2)	0.7
Female (*n* = 26)	9 (34)		
**Child age**			
<36 months (*n* = 47)	15 (32)	1 (0.4–2.6)	0.9
>36 months (*n* = 35)	11 (31)		
**Mother’s age**			
<35 years (*n* = 47)	14 (30)	0.8 (0.3–2)	0.6
>35 years (*n* = 35)	12 (34)		
**Other brothers and sisters**			
Yes (*n* = 42)	14 (33)	1.1 (0.4–2.9)	0.7
No (*n* = 40)	12 (30)		
**Low birth weight**			
Yes (*n* = 36)	12 (33)	0.8 (0.3–2)	0.6
No (*n* = 46)	14 (30)		
**Mother’s school education**			
≤8 years (*n* = 35)	16 (46)	3.1 (1.1–9.1)	0.02 *
>8 years (*n* = 47)	10 (21)		
**Mother’s job**			
Housewife (*n* = 57)	20 (35)	1.7 (0.5–5.7)	0.3
Outside home (*n* = 25)	6 (24)		
**Hearing loss severity**			
Mild (*n* = 22)	4 (18)		
Severe (*n* = 16)	6 (37)		
Deep (*n* = 44)	16 (36)	0.4 (0.1–1.5)	0.1
**Connexin 26 homozygosis**			
Yes (*n* = 19)	3 (16)	0.35 (0.07–1.5)	0.1
No (*n* = 52)	18 (35)		
**Hearing loss familiarity**			
Yes (*n* = 17)	7 (41)	1.7 (0.5–6.0)	0.3
No (*n* = 59)	17 (29)		
**Neonatal intensive care unit (NICU)**			
Yes (*n* = 36)	14 (39)	1.9 (0.7–5.5)	0.2
No (*n* = 44)	11 (25)		
**Syndromes/dysmorphisms**			
Yes (*n* = 32)	14 (44)	2.4 (0.8–7.0)	0.07
No (*n* = 49)	12 (24.5)		
**Congenital infections**			
Yes (*n* = 9)	6 (67)	5.9 (1.1–39)	0.02 *
No (*n* = 67)	17 (25)		
**Craniofacial anomalies**			
Yes (*n* = 11)	6 (54.5)	3.2 (0.7–15)	0.1
No (*n* = 70)	19 (27)		
**Presence of cognitive delay**			
Yes (*n* = 35)	15 (43)	4.1 (1.2–14.9)	0.01 *
No (*n* = 39)	6 (15)		
**Cochlear implant**			
Yes (*n* = 30)	7 (23)	0.5 (0.02–1.6)	0.2
No (*n* = 52)	19 (36)		
**Months from diagnosis**			
<6 (*n* = 32)	8 (25)	0.6 (0.2–1.7)	0.3
>7 (*n* = 46)	17 (37)		

* = *p* < 0.05, significant test.

**Table 3:** Overall stress adjusted for potentially confounding variables.

Variables	Stress > 85 Centile Number (%)		Odds Ratio (IC 95%)	*p* Values
	**<85 Centile**	**≥85 Centile**		
**Cochlear implant Yes**	23	7		
**Cochlear implant No**	15	15	3.29 (0.96–11.6)	0.05
**Cochlear implant Yes**				
Mother’s school education ≤ 8 years	10	3		
Mother’s school education > 8 years	13	4	1.0 (0.14–7.0)	ns
**Cochlear implant No**				
Mother’s school education ≤ 8 years	9	13	0.17 (0.04–0.69)	0.01
Mother’s school education > 8 years	24	6		

Only children with severe and profound hearing loss were considered; *p* < 0.05, significant test.

## Data Availability

Data available on request.
